# Magnetic silica/graphene oxide nanocomposite supported ionic liquid–manganese complex as a powerful catalyst for the synthesis of tetrahydrobenzopyrans

**DOI:** 10.1038/s41598-023-46629-4

**Published:** 2023-11-07

**Authors:** Farkhondeh Dadvar, Dawood Elhamifar

**Affiliations:** Department of Chemistry, Yasouj University, Yasouj, 75918-74831 Iran

**Keywords:** Heterogeneous catalysis, Synthetic chemistry methodology

## Abstract

A novel magnetic silica/graphene oxide nanocomposite supported ionic liquid/manganese complex (Fe_3_O_4_@SiO_2_-NH_2_/GO/IL-Mn) is prepared, characterized and its catalytic application is investigated. The Fe_3_O_4_@SiO_2_-NH_2_/GO/IL-Mn catalyst was synthesized via chemical immobilization of graphene oxide on Fe_3_O_4_@SiO_2_ nanoparticles followed by modification with ionic liquid/Mn complex. This nanocomposite was characterized by using SEM, TGA, FT-IR, PXRD, EDX, TEM, nitrogen adsorption–desorption, and VSM analyses. The catalytic application of Fe_3_O_4_@SiO_2_-NH_2_/GO/IL-Mn was studied in the synthesis of tetrahydrobenzo[b]pyrans (THBPs) in water solvent at RT. This nanocatalyst was successfully recovered and reused at least eight times without a significant decrease in its activity.

## Introduction

Carbon-based materials are very attractive among chemists due to their high efficiency as a support for different catalysts and also their good conductivity^1–3^. One of the most important allotropes of carbon is graphene oxide (GO)^4,5^ which has a two-dimensional and single-layer structure and involves hydroxyl, carboxylic acid, and epoxy groups on its surface^6,7^. The properties of graphene oxides, such as very good specific surface area, biocompatibility, high flexibility, and lightness, endow them with strong potential for applications in catalytic processing^8–10^. However, GO accumulates in salt solutions and biological media. Therefore, to overcome this problem and also for easy separation of GO, recently, the immobilization of graphene oxide on magnetic nanoparticles has been considered^11,12^. In fact, the unique properties of magnetic NPs such as high surface area, availability, easy separation, and recoverability from the environment, make them attractive candidates to composite with GO. Some reports in this matter are TiO_2_/Fe_3_O_4_/GO^13^, Ag_3_PO_4_-Fe_3_O_4_-GO^14^, PEG/Fe_3_O_4_/GO-NH_2_^15^_,_ Fe_3_O_4_/GO-COOH^16^, Fe_3_O_4_/GO/CS^17^, MOF@Fe_3_O_4_@GO^18^, Fe_3_O_4_-GO-(o-MWCNTs)hybrid^19^, Fe_3_O_4_/GO/chitosan^20^ and γ-PGA-Fe_3_O_4_-GO-(o-MWCNTs)^21^. Moreover, several organic functional groups have also been used to modify GO for practical applications^22^. Some reported examples in this matter are GO@IL/MoO_2_(acac)_2_^23^, Cu–NiAAPTMS@GO^24^, GO@melamine^25^, plydopamine@GO/cellulose^26^, Al_2_O_3_/GO cellulose^27^, GO-TCT-DETA^28^, and Mn-UiO-66@GO-NH_2_^29^.

An important process in chemistry is multicomponent reaction (MCR), in which at least three starting materials are used to synthesis valuable organic compounds^30–32^. As example, this process has been effectively used for the synthesis of tetrahydrobenzo[b]pyrans (THBPs)^33,34^ with excellent biological activities such as antiviral, anticancer, and dementia^35,36^. Although, to date, many catalytic systems have been used for the synthesis of THBPs, however, the most of them suffer from drawbacks of high catalyst loading, the use of toxic organic solvents, high reaction temperature, and non-recoverability of the catalyst. Therefore, the preparation of a novel and powerful catalytic system to overcome the aforementioned limitations is an important challenge in this matter.

In view of the above, herein, we report the synthesis and characterization of a novel magnetic silica/graphene oxide nanocomposite supported ionic liquid/Mn complex (Fe_3_O_4_@SiO_2_-NH_2_/GO/IL-Mn). This is effectively applied as an efficient and recoverable catalyst in the synthesis of THBPs.

## Experimental section

### Preparation of Fe_3_O_4_@SiO_2_-NH_2_

For the synthesis of Fe_3_O_4_@SiO_2_-NH_2_, firstly, Fe_3_O_4_ nanoparticles were prepared according to a known method^37^. Then, 0.5 g of Fe_3_O_4_ was added in a solution containing 30 mL of ethanol, 20 mL of distilled water, and 10 mL of ammonia (25%). After that, 70 μL of 3-aminopropyltriethoxysilane (APTES) and 70 μL of tetraethoxysilane (TEOS) were added and the resulted mixture was stirred at 35 °C for 3 h. Finally, the product was separated by using a magnet, washed with distilled water and ethanol, dried at 75 °C for 7 h and denoted as Fe_3_O_4_@SiO_2_-NH_2_.

### Preparation of Fe_3_O_4_@SiO_2_-NH_2_/GO

The Fe_3_O_4_@SiO_2_-NH_2_/GO nanocomposite was prepared as follows. First, 0.3 g of GO was suspended in 20 mL of distilled water for 10 min. Then, 0.5 g of Fe_3_O_4_@SiO_2_-NH_2_ was added and the obtained mixture was vigorously stirred at 70 °C for 2 h. Finally, the product was separated by using a magnet, washed with distilled water and ethanol, dried at 75 °C for 7 h and denoted as Fe_3_O_4_@SiO_2_-NH_2_/GO.

### Preparation of Fe_3_O_4_@SiO_2_-NH_2_/GO/IL

For the preparation of Fe_3_O_4_@SiO_2_-NH_2_/GO/IL, firstly, 1 g of Fe_3_O_4_@SiO_2_-NH_2_/GO nanocomposite was suspended in 50 mL of toluene and sonicated for 20 min at RT. Then, 0.2 mmol of 1-methyl-3-(3-trimethoxysilylpropyl)imidazolium chloride (Im) was added and the obtained mixture was stirred under reflux conditions for 24 h. The product was separated by using a magnet, washed with ethanol, dried at 70 °C for 6 h and denoted as Fe_3_O_4_@SiO_2_-NH_2_/GO/IL.

### Preparation of Fe_3_O_4_@SiO_2_-NH_2_/GO/IL-Mn

For this, 1 g of Fe_3_O_4_@SiO_2_-NH_2_/GO /IL was dispersed in 20 mL of DMSO under ultrasonic irradiation. Then, 0.5 mmol of Mn(OAc)_3_.4H_2_O salt was added and the resulting mixture was stirred at 80 °C for 2 h. The product was separated by using a magnet, washed with ethanol, dried at 70 °C for 6 h and denoted as Fe_3_O_4_@SiO_2_-NH_2_/GO/IL-Mn.

### Synthesis of THBPs using Fe_3_O_4_@SiO_2_-NH_2_/GO/IL-Mn nanocatalyst

For this purpose, the Fe_3_O_4_@SiO_2_-NH_2_/GO/IL-Mn catalyst (0.8 mol%), malononitrile (1 mmol), benzaldehyde (1 mmol) and dimedone (1 mmol) were added in distilled water (10 mL). The resulting mixture was vigorously stirred at RT. The progress of the reaction was monitored by using TLC. After the completion of the reaction, the catalyst was separated by using a magnet. Then, ethyl acetate (20 mL) was added to the residue and the obtained mixture was washed three times with water in a decanter to remove some impurities. Finally, the obtained ethyl acetate solution was placed in an ice bath to crystalize/precipitate the desired pure products.

### IR, ^1^H-NMR and ^13^C-NMR data of THBPs

#### 2-Amino-4-(3-nitrophenyl)-7,7-dimethyl-5-oxo-6,6,8,8-tetrahydro-4*H*-chromene-3-carbonitrile

White solid; yield: 85%; M. P.: 211–212 °C (210–212^35^), IR (KBr, cm^−1^): 3420, 3339 (NH_2_, stretching vibration), 3181 (= C–H, stretching vibration sp^2^), 2958 (C–H, stretching vibration sp^3^), 2186 (CN, stretching vibration), 1673 (C=O, stretching vibration), 1604, 1488 (C=C, Ar stretching vibration sp^2^), 1245 (C–O, stretching vibration). ^1^H-NMR (300 MHz, CDCl_3_): *δ* (ppm) 0.99 (s, 3H), 1.09 (s, 3H), 2.15 (d, 1H, *J* = 15 Hz), 2.33 (d, 1H, *J* = 15 Hz), 2.59 (s, 2H), 4.46 (s, 1H), 7.24 (s, 2H), 7.63–7.75 (m, 2H), 8.2 (s, 1H), 8.3 (d, 1H, *J* = 9 Hz). ^13^C-NMR (75 MHz, CDCl_3_): *δ* (ppm) 27.6, 28.7, 32.5, 35.9, 40.4, 50.5, 56.9, 112.4, 120.1, 121.2, 122.3, 130.1, 134.8, 147.3, 148.7, 159.5, 164.1, 196.1.

#### 2-Amino-4-(4-methylyphenyl)-7,7-dimethyl-5-oxo-6,6,8,8-tetrahydro-4*H*chromene-3-carbonitrile.

White solid; yield: 85%; M. P.: 217–219 °C (218–220^38^), IR (KBr, cm^−1^): 3424, 3328 (NH_2_, stretching vibration), 3036 (=C–H, stretching vibration sp^2^), 2960 (C–H, stretching vibration sp^3^), 2192 (CN, stretching vibration), 1670 (C=O, stretching vibration), 1561, 1471 (C=C, Ar stretching vibration sp^2^), 1241 (C–O, stretching vibration).^1^H-NMR (300 MHz, CDCl3):1.08 (s, 3H), 1.15 (s, 3H), 2.10 (d, 1H, J = 6 MHz), 2.25 (d, 1H, J = 15.2 MHz), 2.25 (s, 3H), 2.52 (s, 2H), 4.43 (s, 1H), 7.05–7.14 (m, 4H), 7.28 (s, 2H) ^13^C-NMR (75 MHz, CDCl3): δ (ppm) 21.2, 27.9, 29.1, 33.1, 35.1, 41.2, 50.9, 64.1, 114.2, 118.7, 127.5, 129.4, 137.0, 140.2, 157.4, 161.5, 196.0

#### 2-Amino-4-(4-methoxyphenyl)-7,7-dimethyl-5-oxo-6,6,8,8-tetrahydro-4H-chromene-3-carbonitrile

White solid; yield: 90%; M. P.: 199–201 °C (196–198^39^), IR (KBr, cm^−1^): 3432, 3332 (NH_2_, stretching vibration), 3100 (=C–H, stretching vibration sp^2^), 2958 (C–H, stretching vibration sp^3^), 2190 (CN, stretching vibration), 1666 (C=O, stretching vibration), 1527, 1419 (C=C, Ar stretching vibration sp^2^), 1249 (C–O, stretching vibration).^1^H-NMR (300 MHz, CDCl3): δ (ppm) 1.05 (s,3H), 1.14 (s, 3H), 2.20 (d, 1H, *J* = 3.4 Hz), 2.23 (d, 1H, *J* = 3.4 Hz), 2.45 (s, 2H), 3.75 (s, 3H), 4.37 (s, 1H), 4.50 (s, 2H, NH2), 6.80 (d, 2H, *J* = 8.6 Hz), 7.15 (d, 2H, *J* = 8.6 Hz). ^13^C NMR (75 MHz, CDCl3): δ (ppm) 27.8, 28.10, 32.4, 34.6, 40.6, 51.2, 63.9, 113.3, 114.6, 115.2, 128.8, 133.5, 135.3, 157.5, 158.4, 161.3, 195.9.

## Results and discussion

**Figure 1:**
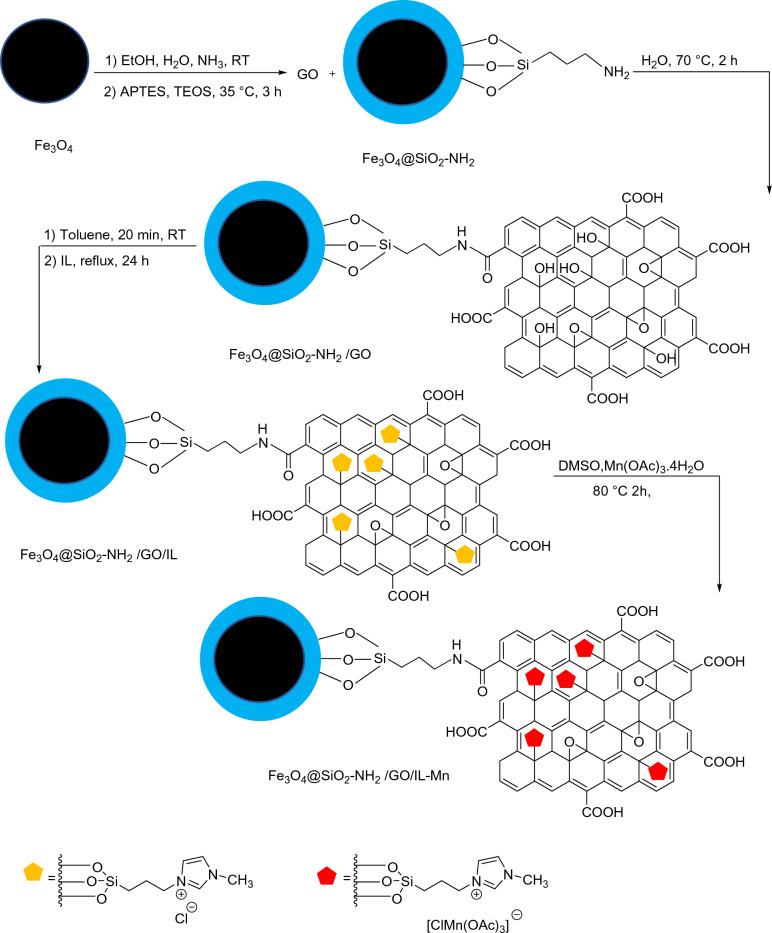
Preparation of the Fe_3_O_4_@SiO_2_-NH_2_/GO/IL-Mn nanocatalyst.

The preparation of the Fe_3_O_4_@SiO_2_-NH_2_/GO/IL-Mn nanocomposite includes four steps (Fig. 1). Firstly, the magnetic Fe_3_O_4_ nanoparticles were modified with TEOS and APTES to give Fe_3_O_4_@SiO_2_-NH_2_ NPs. Secondly, this material was chemically reacted with GO to give Fe_3_O_4_@SiO_2_-NH_2_/GO nanocomposite. Thirdly, the Im-based ionic liquid was chemically grafted on the surface of Fe_3_O_4_@SiO_2_-NH_2_/GO to deliver the Fe_3_O_4_@SiO_2_-NH_2_/GO/IL material. Finally, the last product was treated with manganese acetate to give the Fe_3_O_4_@SiO_2_-NH_2_/GO/IL-Mn nanocatalyst.

**Figure 2:**
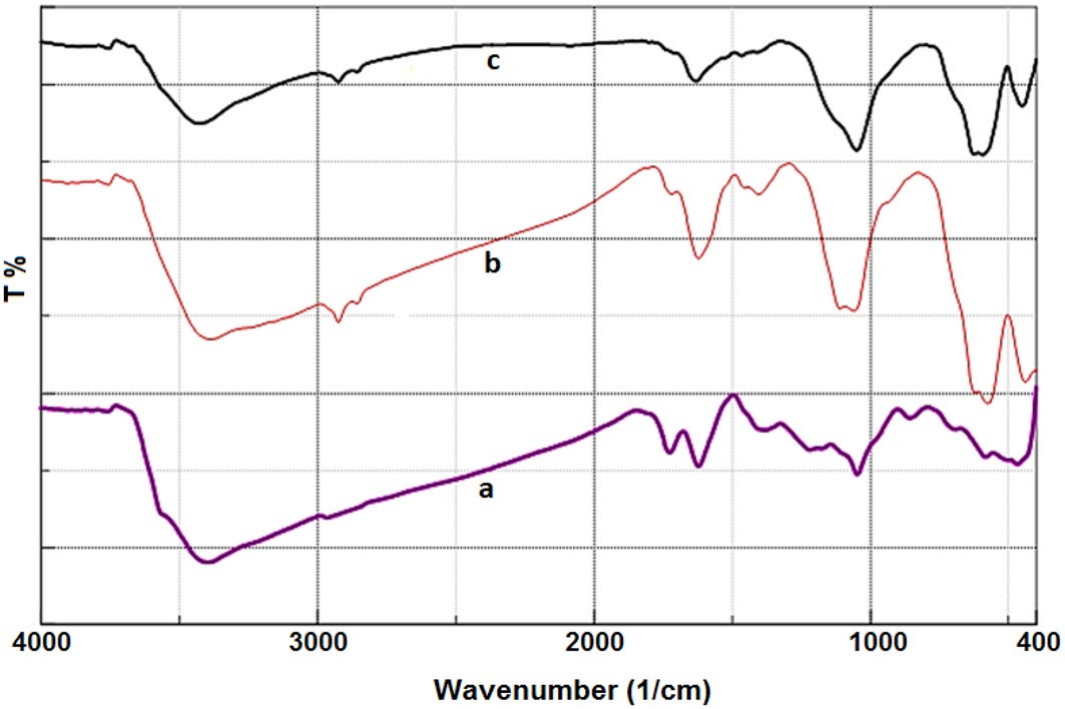
FT-IR spectra of (a) GO, (b) Fe_3_O_4_@SiO_2_-NH_2_ and (c) Fe_3_O_4_@SiO_2_-NH_2_/GO/IL-Mn.

The functional groups of the GO, Fe_3_O_4_@SiO_2_-NH_2_ and Fe_3_O_4_@SiO_2_-NH_2_/GO/IL-Mn materials were determined by using a Fourier transform infrared (FT-IR) spectrometer (Fig. 2). For all samples, the strong peak at 3394 cm^−1^ is due to the O–H bonds of the material surface (Fig. 2a–c)^40^. Moreover, the peaks at 1724, 1519, 1288 and 1049 cm^−1^ are, respectively, associated to carboxyl C=O, aromatic C=C, epoxy C–O and alkoxy C–O bonds of GO (Fig. 2a–c)^41^. For the Fe_3_O_4_@SiO_2_-NH_2_ and Fe_3_O_4_@SiO_2_-NH_2_/GO/IL-Mn materials, the signals at 2825 and 2923 cm^−1^ are attributed to the C–H bonds of the aliphatic groups (Fig. 2b and c)^42^. Moreover, for the latter materials, the peak at 593 cm^−1^ is assigned to the Fe–O bond (Fig. 2b and c)^43^. For Fe_3_O_4_@SiO_2_-NH_2_/GO/IL-Mn, the signal at 1627 cm^−1^ is attributed to C=N bond of ionic liquids (Fig. 2c)^41,44^. In addition, for both Fe_3_O_4_@SiO_2_-NH_2_ and Fe_3_O_4_@SiO_2_-NH_2_/GO/IL-Mn nanomaterials, the strong signals at 1083 and 1215 cm^−1^ are assigned to the Si–O-Si vibrations^45,46^.

**Figure 3:**
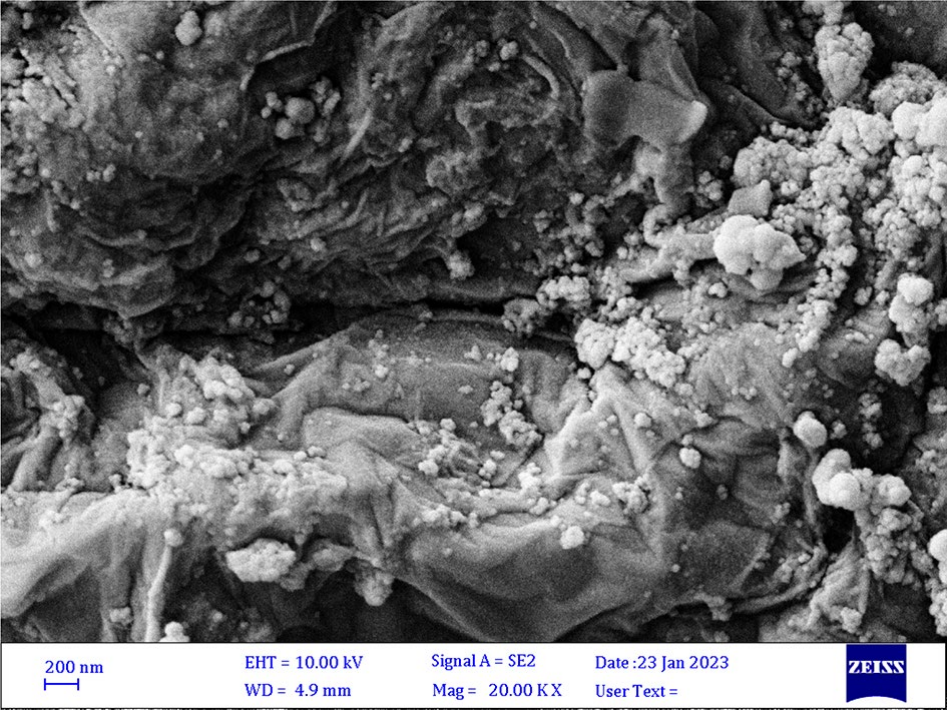
SEM image of Fe_3_O_4_@SiO_2_-NH_2_/GO/IL-Mn.

The surface morphology of Fe_3_O_4_@SiO_2_-NH_2_/GO/IL-Mn was studied by using SEM technique. The spherical nanoparticles of Fe_3_O_4_@SiO_2_ NPs and also the graphene oxide layers were clearly seen in the SEM image (Fig. 3). This confirms the successful formation of the Fe_3_O_4_@SiO_2_-NH_2_/GO composite during applied conditions.

**Figure 4:**
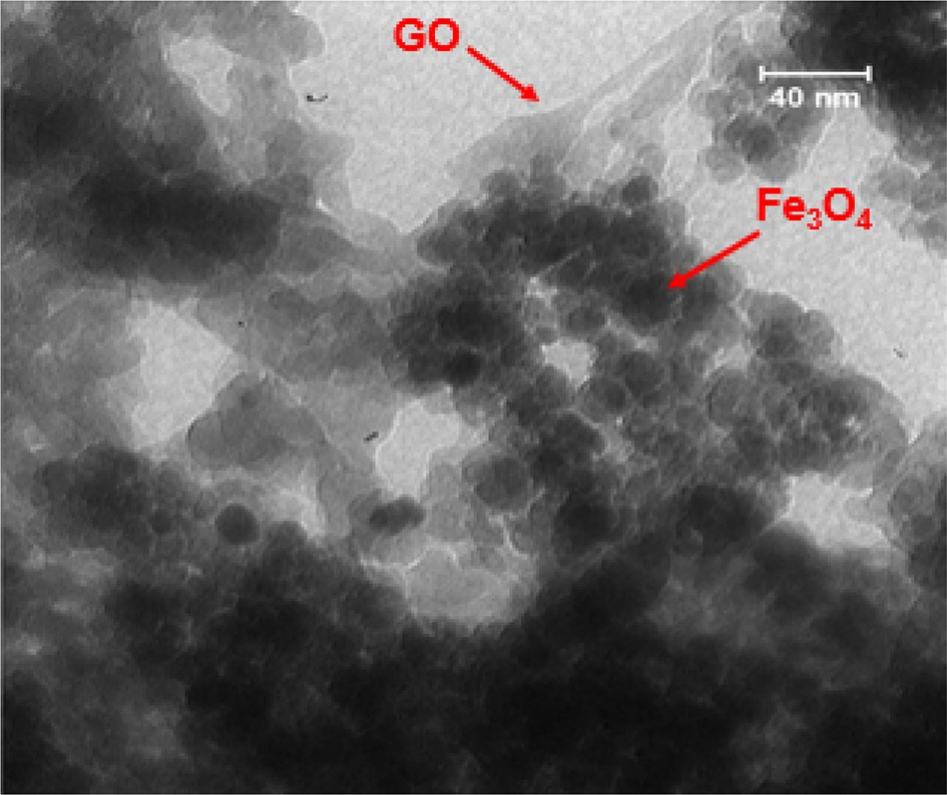
TEM image of Fe_3_O_4_@SiO_2_-NH_2_/GO/IL-Mn.

The TEM analysis of the designed catalyst was also performed to investigate its structure. This analysis showed the catalyst to be composed of spherical Fe_3_O_4_@SiO_2_ NPs and GO layers (Fig. 4).

**Figure 5:**
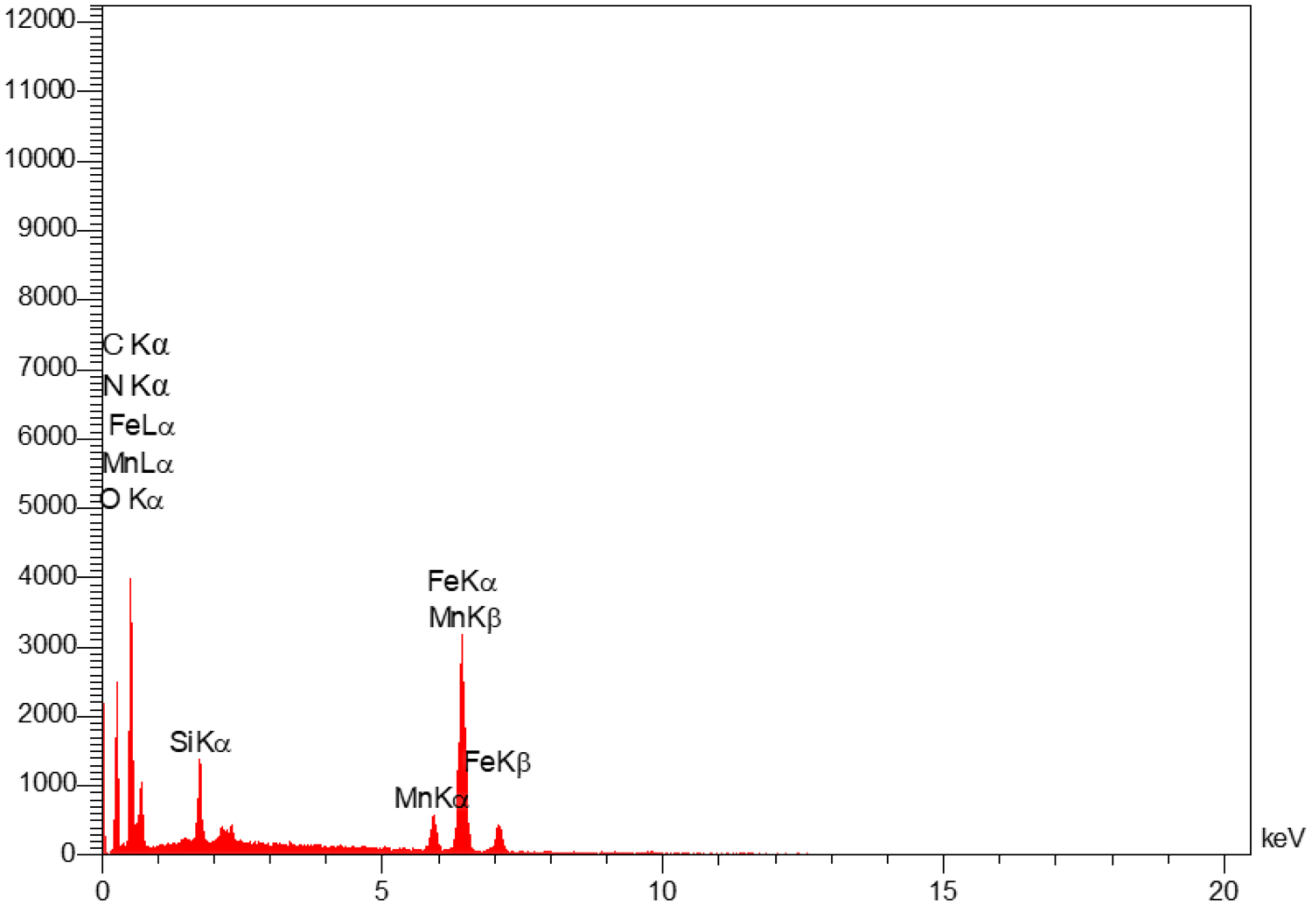
EDX analysis of Fe_3_O_4_@SiO_2_-NH_2_/GO/IL-Mn.

The EDX analysis showed the signals of carbon, nitrogen, oxygen, silicon, manganese and iron elements in the prepared nanocomposite (Fig. 5). This is in good agreement with the FT-IR results, confirming the successful immobilization of IL-Mn complex on Fe_3_O_4_@SiO_2_-NH_2_/GO composite.

**Figure 6:**
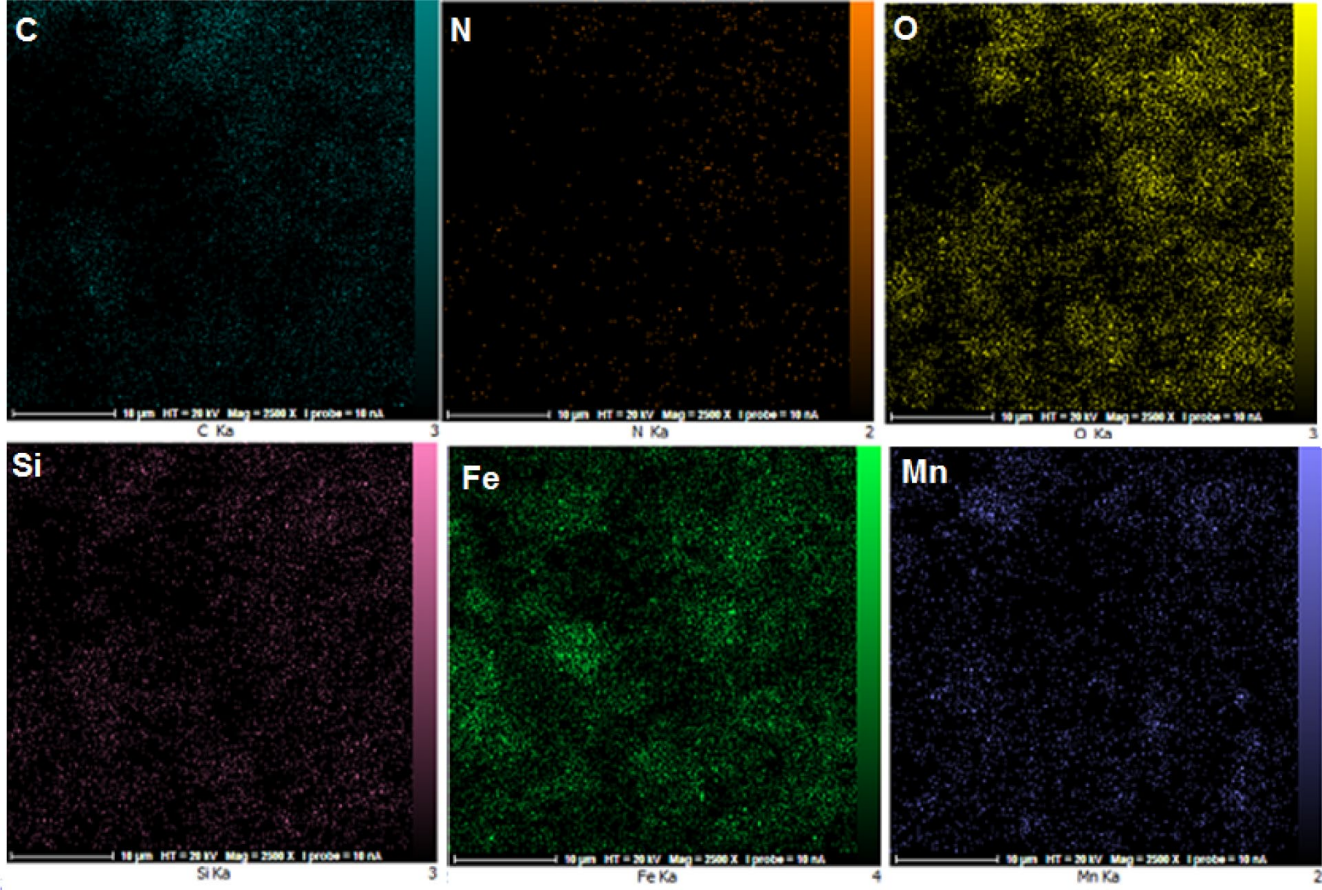
EDX-mapping analysis of the Fe_3_O_4_@SiO_2_-NH_2_/GO/IL-Mn nanocatalyst.

The EDX-mapping analysis of the Fe_3_O_4_@SiO_2_-NH_2_/GO/IL-Mn nanocatalyst is shown in Fig. 6. As seen, all desired elements of C, O, N, Fe, Si and Mn are very well distributed in the material. This is also in good agreement with the FT-IR and EDX results, indicating the successful formation of the designed Fe_3_O_4_@SiO_2_-NH_2_/GO/IL-Mn nanocomposite.

**Figure 7:**
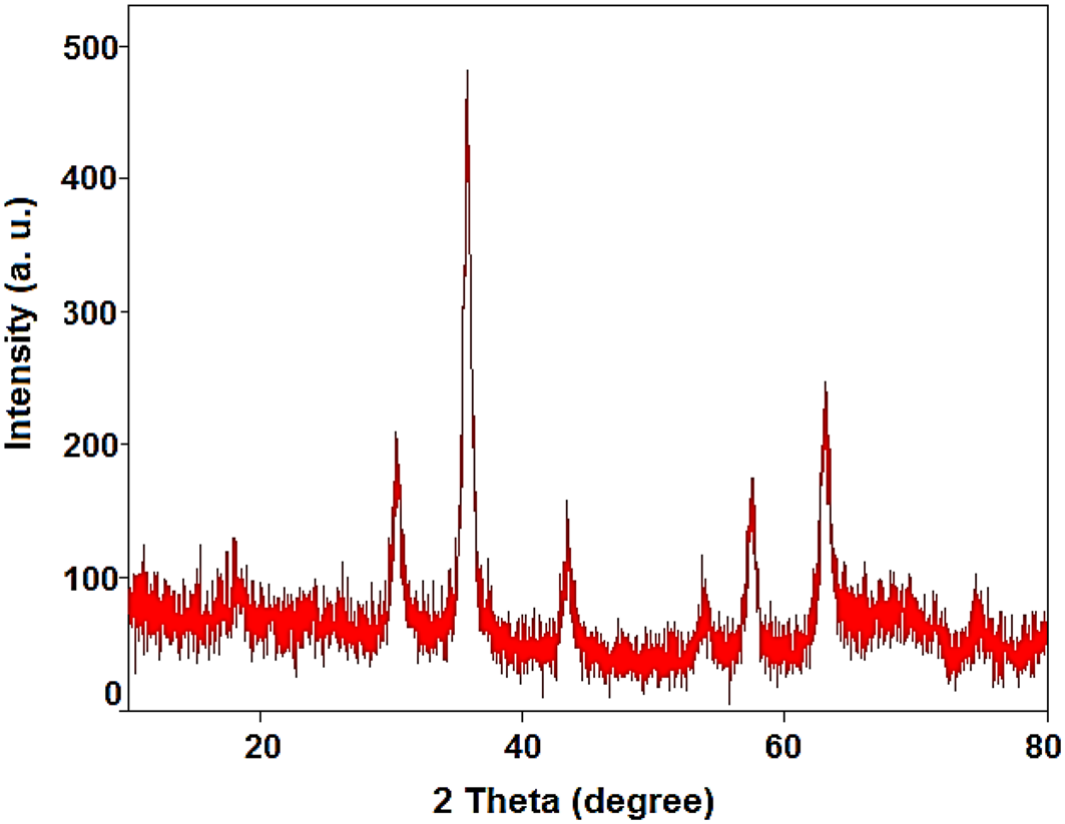
PXRD pattern of the Fe_3_O_4_@SiO_2_-NH_2_/GO/IL-Mn nanocatalyst.

The powder XRD analysis of Fe_3_O_4_@SiO_2_-NH_2_/GO/IL-Mn showed six signals at 2θ of 30, 35.5, 43.1, 54, 57.2, and 63.5 degree, corresponding to the *Miller indices* of 220, 311, 400, 422, 511 and 440, respectively (Fig. 7). These signals are attributed to the spinel structure of magnetic iron oxide NPs,^47,48^ confirming the high stability of the magnetite NPs during modification processes. Also, the peak at 2θ = 19° is related to silica layer of the designed catalyst^49,50^.

**Figure 8:**
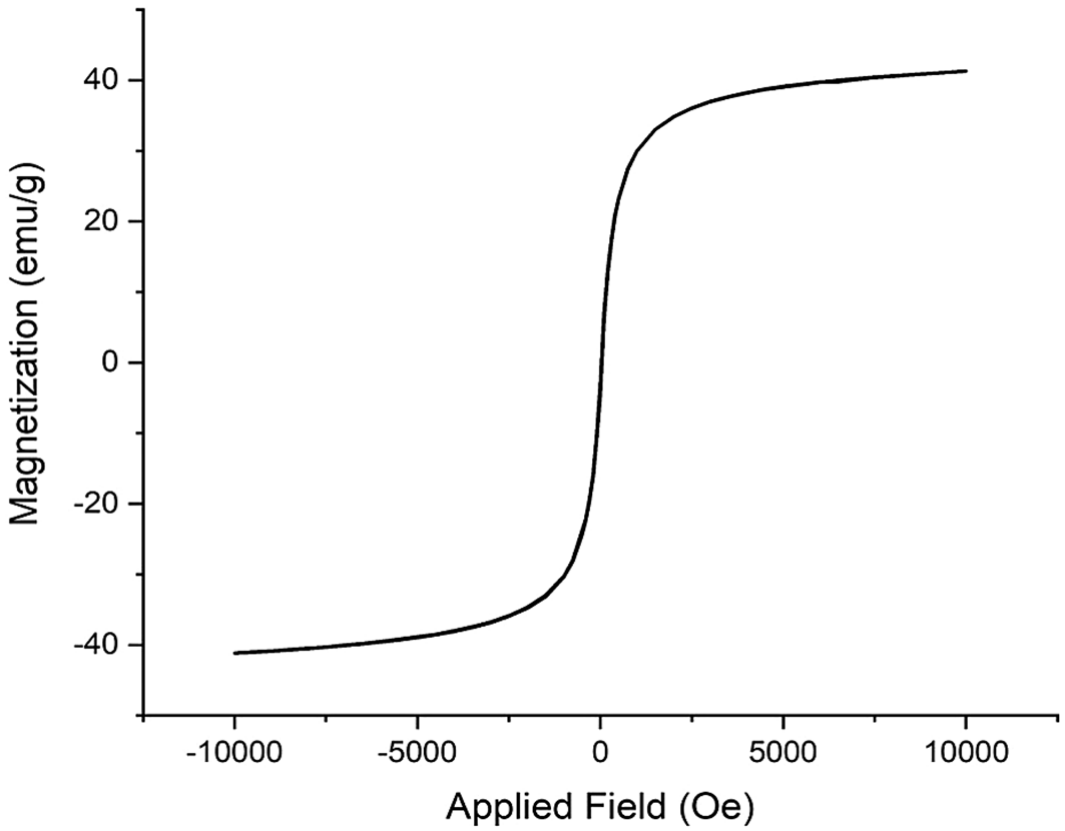
VSM of the Fe_3_O_4_@SiO_2_-NH_2_/GO/IL-Mn nanocatalyst.

According to the VSM analysis, the saturation magnetization of the designed Fe_3_O_4_@SiO_2_-NH_2_/GO/IL-Mn material was found to be 40 emu/g (Fig. 8), confirming its high magnetic properties. This characteristic is very important in the fields of adsorption and catalysis.

**Figure 9:**
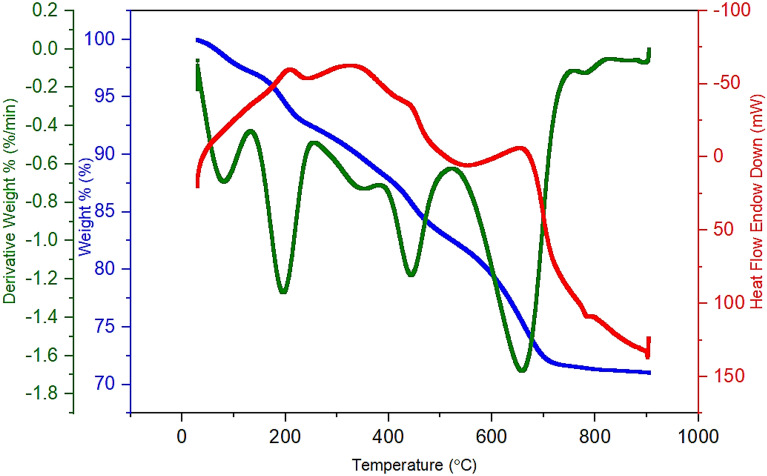
TGA of the Fe_3_O_4_@SiO_2_-NH_2_/GO/IL-Mn nanocatalyst.

Thermal stability of the Fe_3_O_4_@SiO_2_-NH_2_/GO/IL-Mn nanocatalyst was investigated by using thermal gravimetric analysis (TGA, Fig. 9). The first weight loss at temperatures between 10 to 110 °C (3%) is related to the removal of water and alcoholic solvents^39^. The second weight loss at 111–210 °C (4%) is attributed to the removal of the parts of functional groups that are located on the surface of the material. The main weight loss at temperatures more than 220 °C is related to the complete removal of the ionic liquids and also some parts of GO.

**Figure 10:**
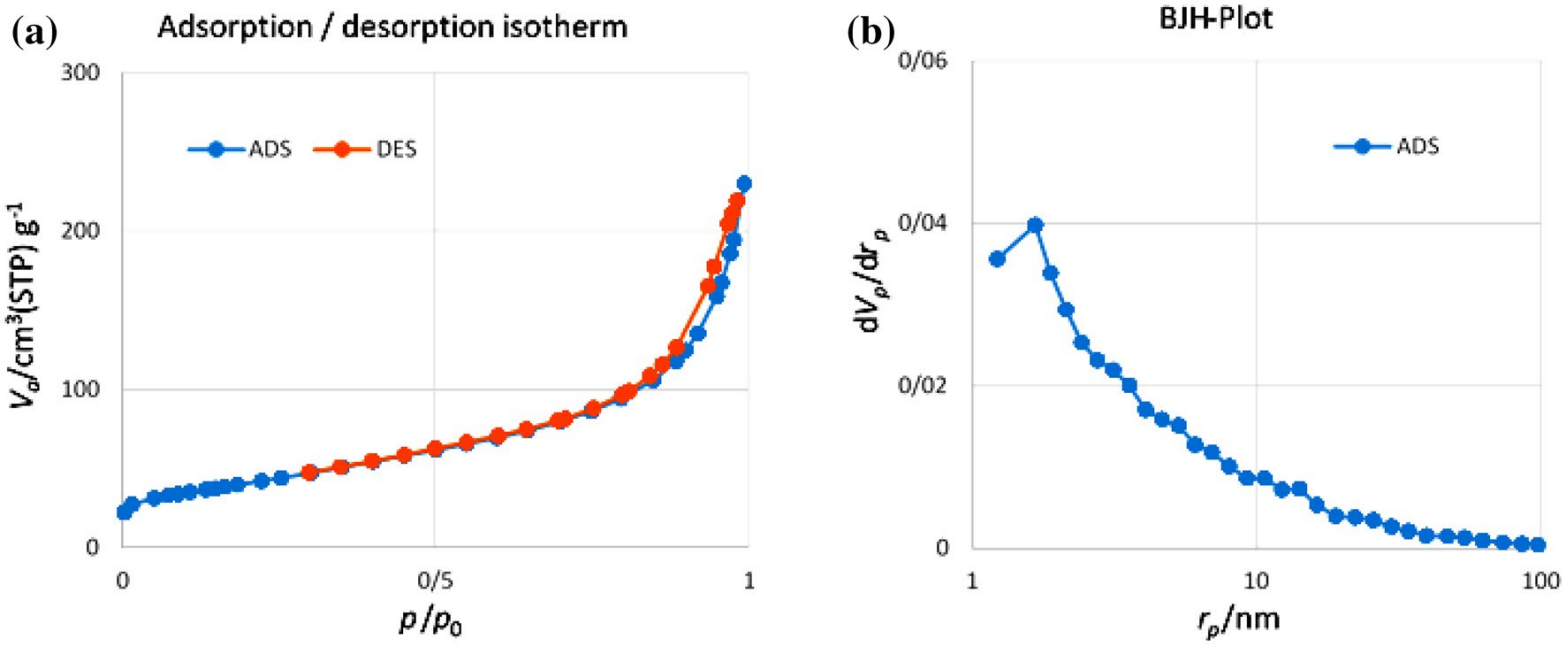
(**a**) Nitrogen adsorption–desorption and (**b**) BJH pore size distribution isotherms of the Fe_3_O_4_@SiO_2_-NH_2_/GO/IL-Mn nanocatalyst.

The nitrogen adsorption–desorption isotherms of the Fe_3_O_4_@SiO_2_-NH_2_/GO/IL-Mn nanocomposite showed a type II curve with a pronounced H3 hysteresis loop, according to the IUPAC classification^51^. The BET specific surface area and total pore volume of the material were calculated to be about 386.5 m^2^/g and 0.35 cm^3^/g, respectively. In addition, the BJH pore size distribution analysis showed a peak with good intensity centered at average pore diameter of about 4.8 nm (Fig. 10).

After preparation and characterization, the catalytic activity of Fe_3_O_4_@SiO_2_-NH_2_/GO/IL-Mn was investigated in the synthesis of THBPs at room temperature (RT). For this, the reaction between benzaldehyde, dimedone and malononitrile was selected as a test model (Table 1). The effect of various parameters such as catalyst loading and solvent was investigated to obtain the best conditions. In the absence of a catalyst, no product was obtained after 3 h, proving the catalyst is necessary for the development of this reaction (Table 1, entry 1). After addition of the catalyst, the reaction was progressed effectively and the best result was obtained in the presence of 0.8 mol% of Fe_3_O_4_@SiO_2_-NH_2_/GO/IL-Mn (Table 1, entries 2–4). It is important to note that increasing the amount catalyst to 1 mol% did not result in a significant change in the reaction yield (Table 1, entry 5). In order to demonstrate the effect of the Mn-centers on the catalytic process, the catalytic activity of Mn-free Fe_3_O_4_@SiO_2_-NH_2_/GO/IL nanocomposite was also investigated. This experiment showed that the Mn-free material gave no yield of the desired product, verifying the process is actually catalyzed by catalytic Mn sites (Table 1, entry 6). This catalytic system was also significantly affected by the solvent. Yields of 58%, 82%, 53% were obtained in toluene, EtOH and also under solvent-free media, respectively. Pleasingly, in water, the best yield was obtained (Table 1, entry 4). Accordingly, 0.8 mol% of catalyst, water solvent and RT were identified as the optimal conditions (Table 1, entry 4).

**Table 1:** Effect of solvent and catalyst loading in the synthesis of THBPs at RT.


Entry	Catalyst (mol%)	Solvent	Time (min)	Yield (%)
1	–	H_2_O	180	–
2	Fe_3_O_4_@SiO_2_-NH_2_/GO/IL-Mn (0.4)	H_2_O	40	43
3	Fe_3_O_4_@SiO_2_-NH_2_/GO/IL-Mn (0.6)	H_2_O	40	72
4	Fe_3_O_4_@SiO_2_-NH_2_/GO/IL-Mn (0.8)	H_2_O	40	95
5	Fe_3_O_4_@SiO_2_-NH_2_/GO/IL-Mn (1)	H_2_O	40	96
6	Fe_3_O_4_@SiO_2_-NH_2_/GO/IL (0.01 g)	H_2_O	40	12
7	Fe_3_O_4_@SiO_2_-NH_2_/GO/IL-Mn (0.8)	Toluene	40	58
8	Fe_3_O_4_@SiO_2_-NH_2_/GO/IL-Mn (0.8)	EtOH	40	82
9	Fe_3_O_4_@SiO_2_-NH_2_/GO/IL-Mn (0.8)	Solvent-free	40	53

With the optimum conditions in hand, various aldehyde derivatives containing both electron withdrawing and electron donating substituents were used as substrate (Table 2). All of these aldehydes delivered the desired products in high yield at short time. It was also found that Fe_3_O_4_@SiO_2_-NH_2_/GO/IL-Mn offers high turnover number (TON) and turnover frequency (TOF) for all products, confirming the high ability of the present catalytic system to synthesis a wide range of biologically active THBPs.

**Table 2:** Synthesis of THBPs in the presence of Fe_3_O_4_@SiO_2_-NH_2_/GO/IL-Mn at RT.


Entry	R	Time (min)	TON^a^	TOF^b^	Yield (%)	Found M. P. (°C)	Reported M. P. (°C)
1	H	40	118.75	179.92	95	221–224	222–224^52^
2	4-Cl	26	112.5	261.62	90	210–213	210–212^35^
3	4-OMe	35	112.5	193.96	90	199–201	196–198^39^
4	4-Me	37	106.25	174.18	85	217–219	218–220^38^
5	3-NO_2_	20	106.25	321.97	85	211–212	210–212^35^
6	4-NO_2_	15	110	440	88	181–183	182–184^35^

^a^Turnover number [defined as yield (%)/cat. (mol%)]. ^b^Turnover frequency [defined as TON/reaction time (h)].

**Figure 11:**
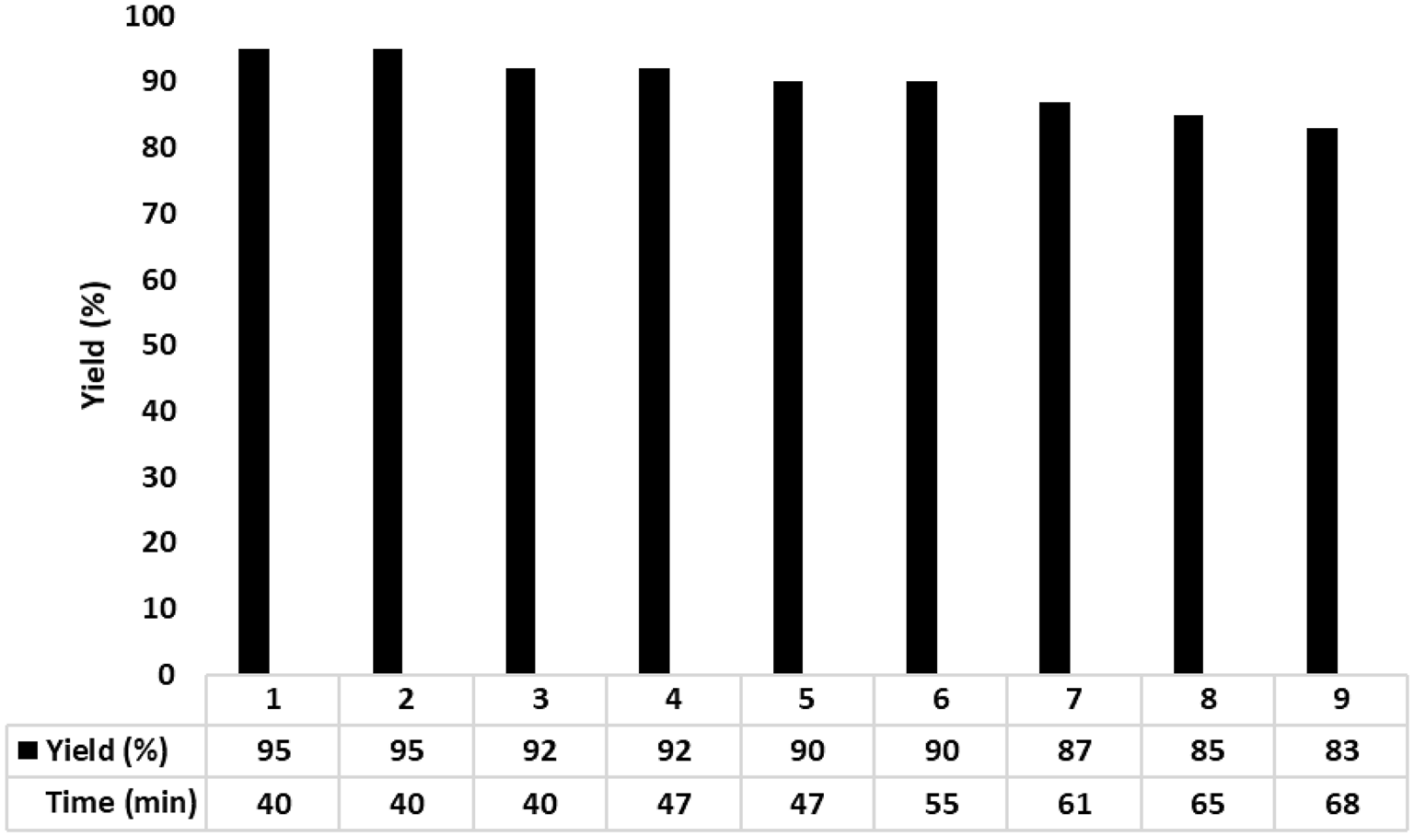
Recoverability and reusability of Fe_3_O_4_@SiO_2_-NH_2_/GO/IL-Mn.

The recoverability and reusability of Fe_3_O_4_@SiO_2_-NH_2_/GO/IL-Mn were also investigated in the reaction model. For this, after finishing of the reaction, the catalyst was easily separated by using a magnet. Then, it was reused in the next run under the same conditions as the first run. These steps were repeated and it was found that the catalyst could be recovered and reused for at least eight times with no significant decrease in efficiency (Fig. 11). These findings confirm high performance and very good stability of the designed catalyst under applied conditions.

Next, a leaching test was performed in the model reaction to investigate the nature of the Fe_3_O_4_@SiO_2_-NH_2_/GO/IL-Mn nanocatalyst under the applied conditions. For this, after the conversion was about 45% complete, the catalyst was magnetically removed. Then, the progress of catalyst-free residue was monitored. Interestingly, after 120 min, no notable conversion was observed. This proves no leaching of Mn species in the reaction solution under the applied conditions and also the heterogeneous nature of the designed catalyst.

Furthermore, the reactivity of the catalyst was investigated under optimal conditions. For this purpose, the model reaction was carried out and its progress was monitored using TLC. After the completion of the reaction, the starting materials were again added to the reaction vessel in the same proportion as the first run. These steps were repeated and the results showed that the activity of the Fe_3_O_4_@SiO_2_-NH_2_/GO/IL-Mn nanocatalyst is maintained for at least seven runs without a significant decrease in performance (Table 3).

**Table 3:** Catalytic reactivity of the Fe_3_O_4_@SiO_2_-NH_2_/GO/IL-Mn nanocomposite.

Run	1	2	3	4	5	6	7	8
Time (min)	40	40	47	50	53	60	60	64

**Figure 12:**
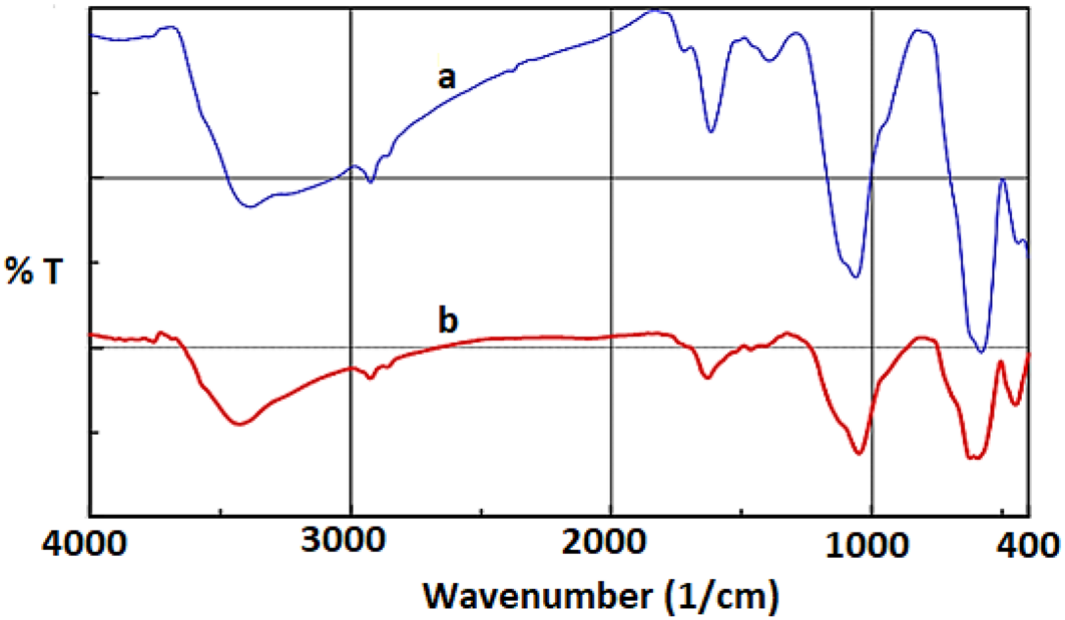
FT-IR spectra of (a) fresh Fe_3_O_4_@SiO_2_-NH_2_/GO/IL-Mn and (b) recovered Fe_3_O_4_@SiO_2_-NH_2_/GO/IL-Mn.

In the next, in order to study the chemical and structural stability of the catalyst under applied conditions, the FT-IR and XRD analyses of the recovered catalyst were performed after fifth run. As shown in Fig. 12, the FT-IR spectrum of the recovered Fe_3_O_4_@SiO_2_-NH_2_/GO/IL-Mn showed a pattern similar to the FT-IR of fresh nanocatalyst, proving the high stability of the designed material under the applied reaction conditions.

**Figure 13:**
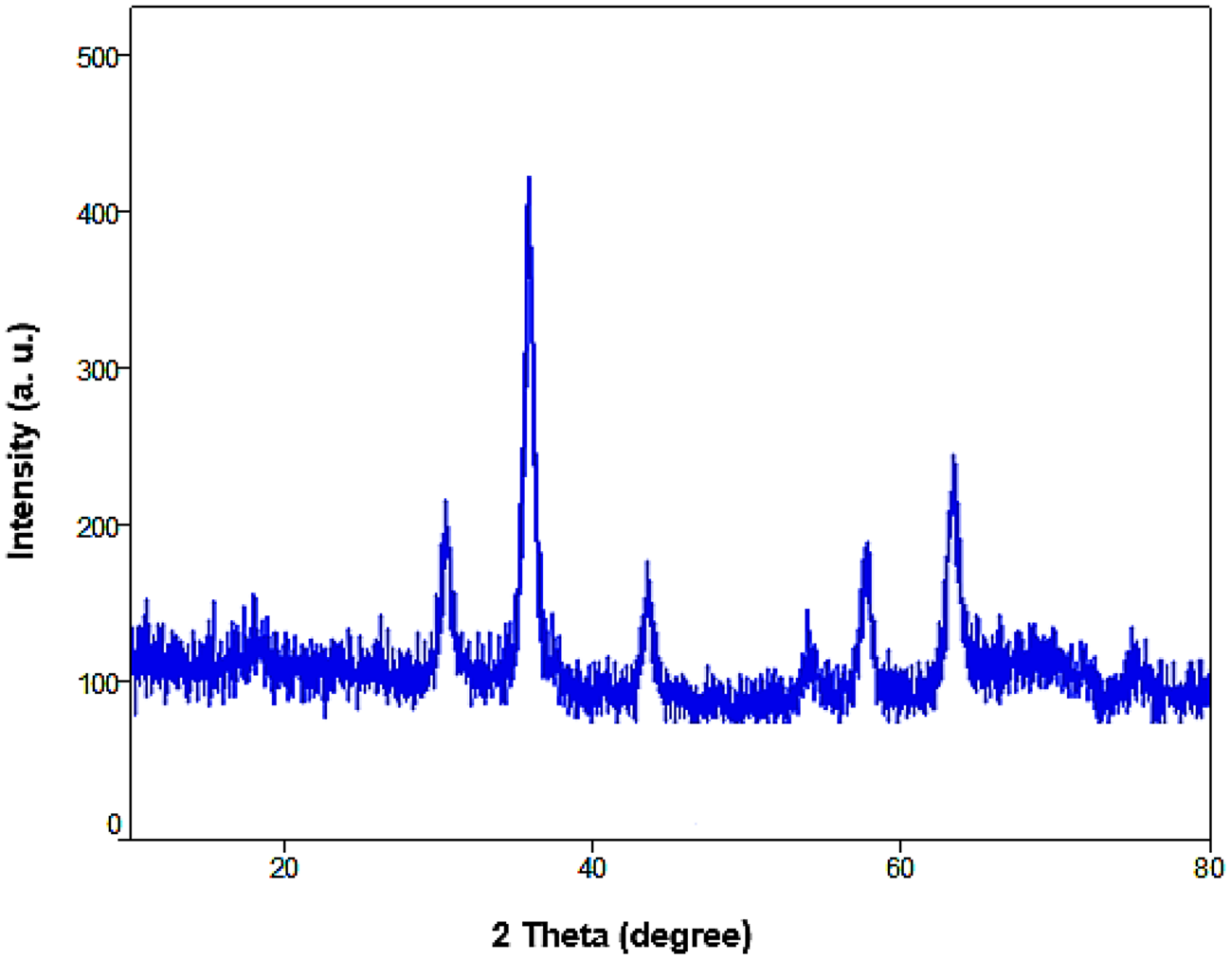
PXRD pattern of the recovered Fe_3_O_4_@SiO_2_-NH_2_/GO /IL-Mn nanocomposite.

The PXRD of the recovered Fe_3_O_4_@SiO_2_-NH_2_/GO/IL-Mn also illustrated six peaks at 2θ of 30, 35.5, 43.1, 54, 57.2, and 63.5, which are in good agreement with the PXRD pattern of the fresh nanocatalyst, proving the high stability of the crystalline structure of Fe_3_O_4_ NPs during the reaction process (Fig. 13).

Finally, the performance of Fe_3_O_4_@SiO_2_-NH_2_/GO/IL-Mn nanocomposite was compared with some previous catalytic systems in the synthesis of THBPs (Table 4). The results showed that our catalyst is better in terms of reaction conditions, catalyst loading and recovery times. These findings may be attributed to the magnetic nature of Fe_3_O_4_@SiO_2_-NH_2_/GO/IL-Mn as well as the positive effect of chemically immobilized ionic liquids in the stabilization of the catalytically active Mn-species.

**Table 4:** The comparative study of Fe_3_O_4_@SiO_2_-NH_2_/GO/IL-Mn with previously reported catalysts.

Catalyst	Conditions	Recovery times	Ref.
MGO-D‐NH‐(CH_2_)_4_‐SO_3_H	Cat.0.02 g, H_2_O/ethanol, 35 ºC	6	^53^
Fe_3_O_4_@SiO_2_@TiO_2_	Cat. 0.01 g, solvent free, 100 ºC	6	^54^
[Et_3_NH][HSO_4_]	Cat. 0.025 g, solvent free/MW	3	^55^
CaO@SiO_2_-SO_3_H	Cat. 0.02 g, H_2_O/50 ºC	6	^56^
GO–Si–NH_2_–PMo	Cat. 0.04 g, solvent-free 90 ºC	5	^57^
Fe_3_O_4_@SiO_2_-NH_2_/GO/IL-Mn	Cat. 0.8 mol%. H_2_O/RT	8	This work

**Figure 14:**
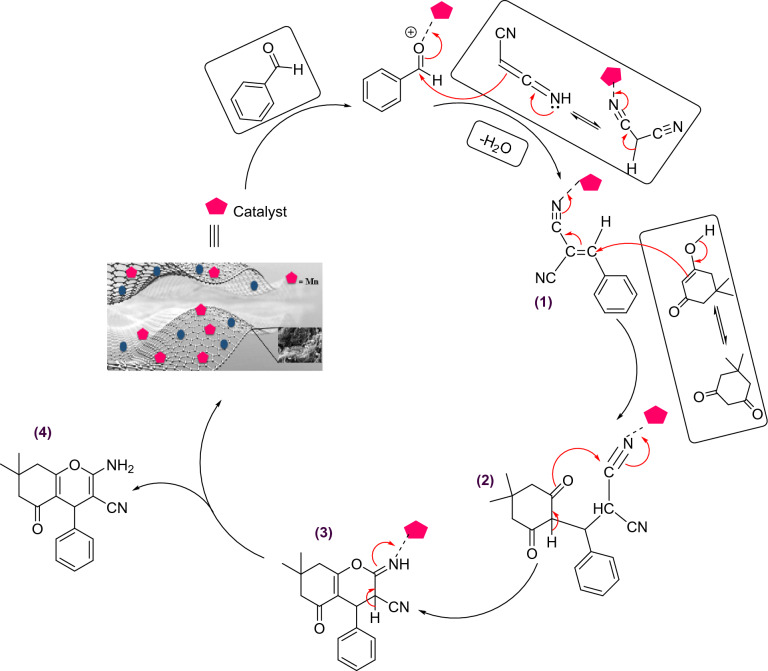
Proposed mechanism for the synthesis of THBPs using the Fe_3_O_4_@SiO_2_-NH_2_/GO/IL-Mn nanocomposite.

A plausible mechanism for the synthesis of THBPs using Fe_3_O_4_@SiO_2_-NH_2_/GO/IL-Mn is outlined in Fig. 14. At first, the malononitrile and the Mn-activated aldehyde are condensed through the Knoevenagel condensation to give intermediate **1**. Intermediate **2** is then delivered via a Michael-type addition between the enol form of dimedone and intermediate **1**. An intramolecular cyclo-condensation is performed on intermediate **2** to give intermediate **3**. Finally, the intermediate **3** is converted to the desired product **4** through a tautomerization process^58^.

## Conclusion

In this study, for the first time, a manganese-containing IL-modified Fe_3_O_4_@SiO_2_-NH_2_/GO nanocomposite was prepared, characterized and used as a novel catalyst for the synthesis of THBPs. The high chemical and thermal stability of the designed catalyst were confirmed by using FT-IR, TGA and EDX analyses. The PXRD and VSM analyses showed high magnetic properties of the designed catalyst. The SEM and TEM analyses also confirmed the successful formation of the Fe_3_O_4_@SiO_2_-NH_2_/GO composite. The Fe_3_O_4_@SiO_2_-NH_2_/GO/IL-Mn catalyst was effectively used in the synthesis of THBPs and gave the desired products in high yields. The leaching test and also the recoverability and reactivity studies clearly showed high performance and stability of the catalyst under applied conditions.

## Data Availability

All data and materials are included in the manuscript.
